# The Subviral RNA Database: a toolbox for viroids, the hepatitis *delta virus *and satellite RNAs research

**DOI:** 10.1186/1471-2180-6-24

**Published:** 2006-03-06

**Authors:** Lynda Rocheleau, Martin Pelchat

**Affiliations:** 1 Department of Biochemistry, Microbiology and Immunology, Faculty of Medicine, University of Ottawa, Ottawa, Ontario, K1H 8M5, Canada

## Abstract

**Background:**

Viroids, satellite RNAs, satellites viruses and the human hepatitis *delta *virus form the 'brotherhood' of the smallest known infectious RNA agents, known as the subviral RNAs. For most of these species, it is generally accepted that characteristics such as cell movement, replication, host specificity and pathogenicity are encoded in their RNA sequences and their resulting RNA structures. Although many sequences are indexed in publicly available databases, these sequence annotation databases do not provide the advanced searches and data manipulation capability for identifying and characterizing subviral RNA motifs.

**Description:**

The Subviral RNA database is a web-based environment that facilitates the research and analysis of viroids, satellite RNAs, satellites viruses, the human hepatitis *delta *virus, and related RNA sequences. It integrates a large number of Subviral RNA sequences, their respective RNA motifs, analysis tools, related publication links and additional pertinent information (ex. links, conferences, announcements), allowing users to efficiently retrieve and analyze relevant information about these small RNA agents.

**Conclusion:**

With its design, the Subviral RNA Database could be considered as a fundamental building block for the study of these related RNAs. It is freely available via a web browser at the URL: .

## Background

Viroids, satellite RNAs, satellites viruses and the human hepatitis *delta *virus (HDV) are the smallest known infectious RNA agents, identified as the Subviral RNAs. The HDV genome consists of a small single-stranded, circular negative sense RNA genome (~1,700 nucleotides, nt) containing self-cleaving motifs (i.e. *delta *ribozymes), and a single open reading frame (ORF) encoding two viral proteins (HDAgs) [1]. HDV requires the hepatitis B virus (HBV) surface antigen for virion assembly and dissemination. Viroids are small non-coding single-stranded circular RNAs (~400 nt) which are unencapsidated and replicate autonomously into host plants [2]. Viroids are grouped into two families based on the presence or not of conserved regions, hammerhead ribozymes and on their subcellular localization (nucleus or chloroplast). Satellites do not possess genes encoding proteins needed for their replication and depend on helper viruses for their multiplication. Satellites include both satellite viruses and satellite nucleic acids. Satellite viruses consist of single-stranded RNA genomes encapsidated by satellite-encoded proteins. Satellite RNAs are a sub-group of satellite nucleic acids and include double-stranded satellite RNAs, and single-stranded satellite RNAs [3]. Double-stranded satellite RNAs are encapsidated by helper virus coat proteins. Single-stranded satellite RNAs possess genome that do not encode a capsid protein, and are classified into three subgroups: large satellite RNAs which encode proteins, small linear satellite RNAs, and circular satellite RNAs (also known as viroid-like satellite RNAs).

Most of the Subviral RNA agents are composed of single-stranded RNA molecules that rely significantly on proteins from their hosts or from helper viruses for replication and propagation [1-3]. In addition, because the genome of viroids and viroid-like satellite RNAs have no coding properties and HDV possesses a very limited coding capacity, it is generally accepted that Subviral RNAs are mosaics of functional RNA motifs providing specific activities (i.e. ribozymes) and signals for recruiting and triggering host proteins (i.e. RY motif; [4]). Extensive molecular research in the field of these agents has produced invaluable sequence data that are being deposited into publicly available databases. However, sequence annotation databases do not provide the advanced searches and data manipulation capabilities to identify and classify Subviral species and their RNA motifs. Consequently, we have designed an online database to facilitate research on Subviral RNA species by presenting a large number of sequences in a user-friendly format [5]. Here we describe the implementation of a web-based environment that is composed of three components (Subviral RNA sequences, RNA motifs, and related information) and that integrates various web-based tools to customize and analyze these sequences and their respective RNA motifs.

## Construction and content

The Subviral RNA Database is hosted on a dedicated server built around a LAMP setup: Linux, Apache, MySQL, Perl (an operating system (OS), a web-server, a database and a script language, respectively). This architecture maximizes the speed and permits a superior customization of the database. In addition, it allows the Subviral server to offer several web-based tools (e.g. Species Identification Service, ClustalW, Blast, Weblogo) and web-forms. Using these forms, users can record directly their entries related to links, conferences and announcements, which are subsequently validated and dynamically displayed using the web interface.

To integrate new data autonomously, automated in-house scripts search for new Subviral RNA sequences, annotations and related publications from the NCBI server. New sequences are then manually adjusted in order to standardize sequences among a specific species (i.e. same sequence origin and same polarity (genomic vs antigenomic) for single-stranded RNA species).

## Utility and discussion

**Figure 1:**
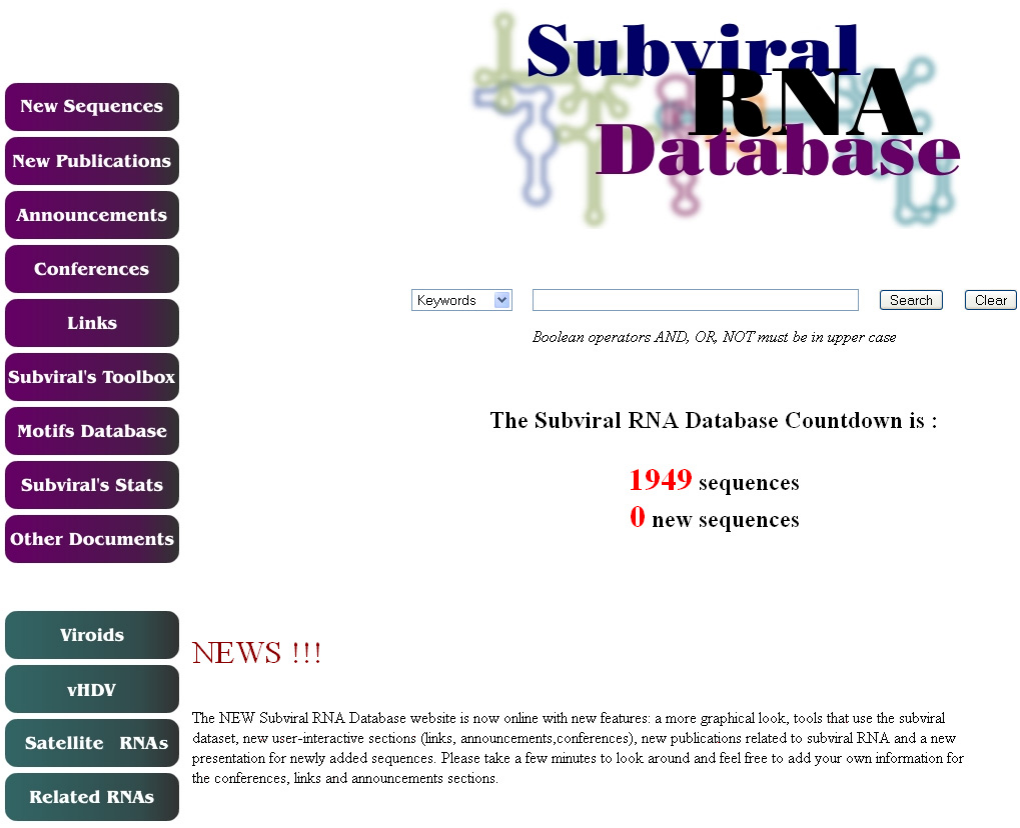
**Organization of the Subviral RNA Database**. Sections related to sequence data, new contents, announcements, conferences, links, bioinformatics tools and Subviral RNA motifs can be accessed directly using buttons located at the left side.

From a simple sequence database [5], the Subviral RNA Database has now evolved into a multi-database system that integrates data on sequences, structural RNA motifs, and related information (links, conferences, announcements, publications). The Subviral RNA Database is presented using a new easy-to-use web interface (Figure 1). Upon entering into the web site, sequences, RNA motifs, new related publication links and relevant information on these RNA agents (ex. laboratory links, conferences, and announcements) can be accessed from the interface. Moreover, a section presenting a collection of statistics and a new search engine are now included. The latter is designed to efficiently search the entire database and to display the queried information.

The four sections related to Subviral RNA sequences form the main part of the database (i.e. Viroids, vHDV, Satellite RNAs, and Related RNAs). All the RNA variants are indexed according to their families, genus and species in a classification scheme based on the criteria established by the International Committee on Taxonomy of Viruses (ICTV) [6]. When not assigned by the ICTV, specific species are classified based on sequence similarities. Selection of a family leads to a section presenting the complete names of the various RNA agents, their abbreviation, the number of sequence variants, their size distribution, the calculated secondary structure of representative variants, and the species decimal code assigned by the ICTV database, when available. For each specific RNA species, users have access to a complete list of the sequence variants and links to identical sequences. Identical sequences for a specific variant could indicate enhanced *in vivo *sequence fitness. In addition, links to known RNA motifs specific for each variant are indicated. From this section, users can select sequences, individually or in groups, and display them under various formats for further analysis using users' software. These entries can also be processed directly on the Subviral server using various bioinformatics tools (see below), which results are either displayed in HTML format or saved in raw text to facilitate transfer to another program.

**Figure 2:**
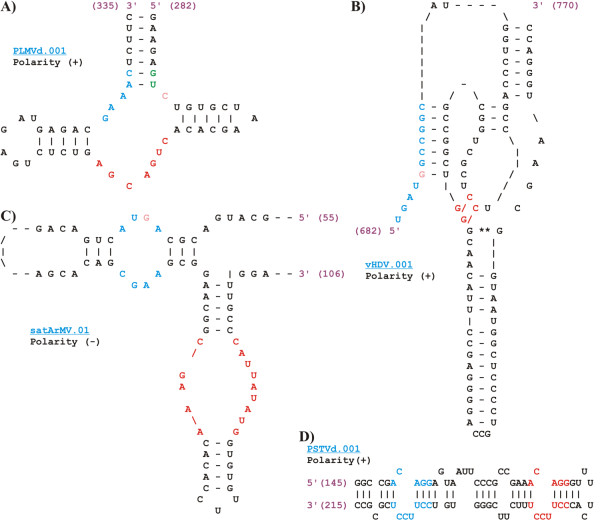
**Overview of the different RNA motifs associated with the indexed Subviral RNA species**. Users have access to information on various RNA motifs found in subviral RNA agents. A) Type III hammerhead ribozyme; B) *delta *ribozyme; C) hairpin ribozyme; and D) RY motif. Important nucleotides for each RNA motif are colored.

**Figure 3:**
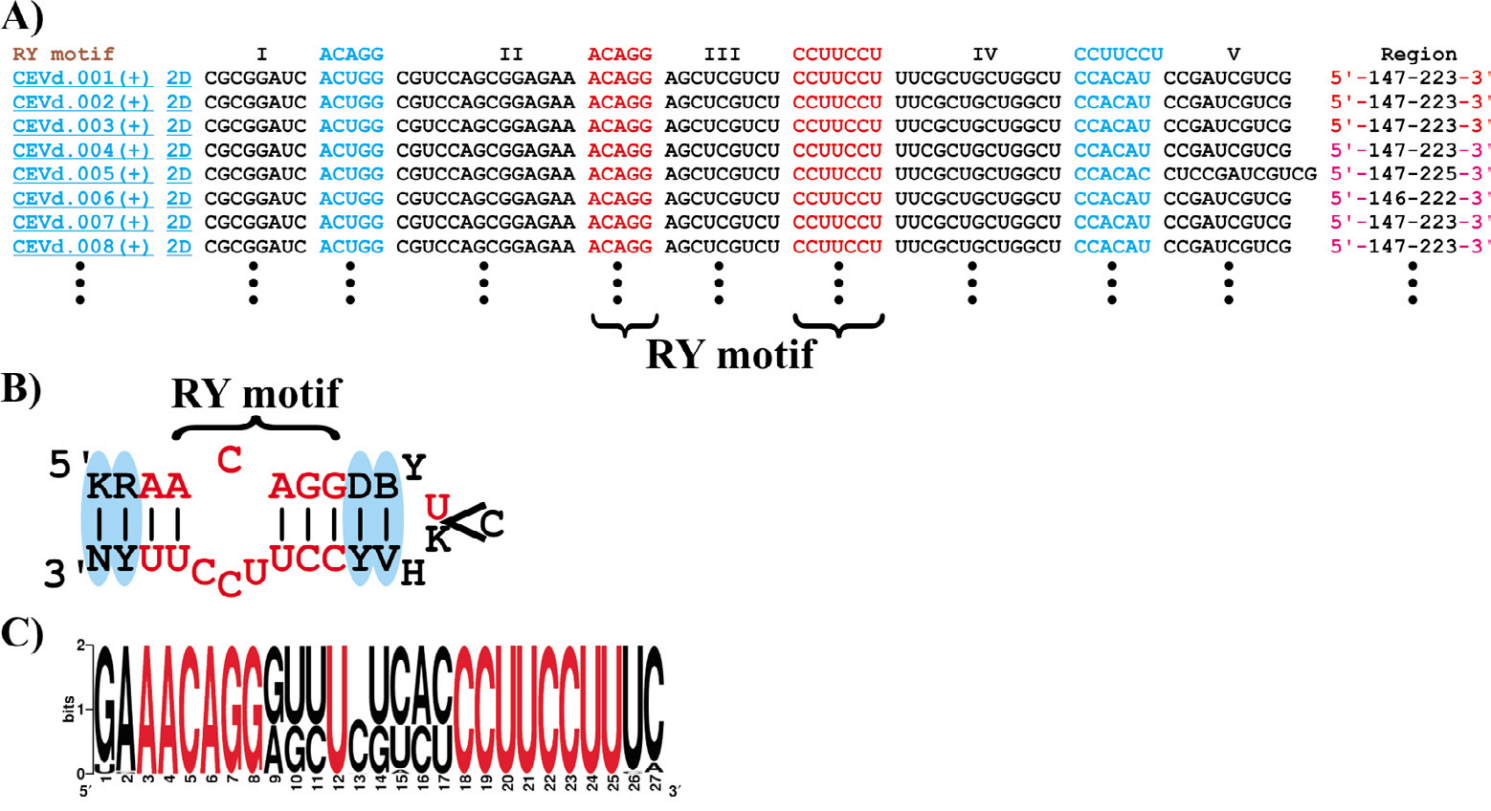
**Analysis of the 260 RY motifs indexed in the Subviral RNA database**. A) Structural representation of the primary sequences of the RY motifs. B) Consensus secondary structure derived from the analysis of natural variants of the RY motifs. Greater that 99% conserved nucleotides are indicated in red, base-pairs conserved through covariations are indicated by blue ovals, and IUPAC 1-letter code abbreviations are used for the identity of the non-conserved nucleotides on the secondary structure (B: U/C/G, D: A/U/G, H: A/U/C, K: U/G, N: A/C/G/U, R: A/G, V: A/C/G, Y: C/U). C) Logo representation of the sequence conservation of the RY motifs [8]. Greater that 99% conserved nucleotides are indicated in red.

Because most Subviral RNAs are mosaics of functional RNA motifs, an RNA motif database presenting the known motifs associated with the indexed species is now included. Most of these RNA motifs were determined by sequence comparison using experimentaly derived motifs as models. The secondary structures of the motifs without pseudoknots were also supported thermodynamically using the Mfold software [7]. Primary sequences of specific motifs can be obtained from structural alignment representation. The secondary structure of each RNA motif indexed in the database can also be displayed dynamically using in-house scripts in plain text or HTML formatted (Figure 2). When HTML is chosen, important nucleotides are indicated by a color code. These information can be used to analyze natural sequence deviations and the variability of the secondary structures for each RNA motif. Figure 3 shows an example of how the database can be used to obtain information on base-pair covariation and on sequence conservation on the 260 RY motifs located near the right terminal stem-loop of a number of viroids of the family *Pospiviroidae *[4]. This analysis clearly shows that nucleotides forming the RY motifs are highly conserved. In contrast, the identity of the surrounding nucleotides is not conserved but the analysis of base-pair covariation suggests a strong selection pressure to maintain this region as double-stranded.

Various bioinformatics web tools such as ClustalW, WebLogo and Blast (which uses the Subviral RNA dataset as a searchable database) are now accessible directly on the Subviral RNA Database server. In addition, in-house tools such as QuickFasta (which offers a quick and easy way to build fasta files from the Subviral dataset) and a Species Identification Service have been implemented. The latter uses a queried sequence and attempts to align it, using ClustalW, to a set of preselected sequences for each species and produces a report including a phylogenetic tree. Finally, sections related to new sequences, new publications, various related links, conferences and announcements can also be accessed through the database.

## Conclusion

At the time of this writing, the Subviral RNA database contains 1949 sequences indexed in 83 species. It comprises information on 1046 viroid sequences, 83 complete and 508 partial HDV sequences, 299 satellites RNA sequences and 13 related RNA sequences. Users have also access to information on 189 type I hammerhead ribozymes, 320 type III hammerhead ribozymes, 8 hairpin ribozymes, 203 *delta *ribozymes and 260 RY motifs. As soon as they will become available, secondary structure predictions of new RNA motifs will also be implemented.

The Subviral RNA Database is designed as a robust and relational database system for these unusual species and their RNA motifs, and also to function as a resource for the entire scientific community by providing full public access to recent sequences, along with many tools to explore and analyze those data. Users of this database are encouraged to provide corrections, or other information, for inclusion in the database via electronic mail mpelchat@uottawa.ca.

## Availability and requirements

This database is publicly accessible by web browser at , is OS independent (Linux, Windows, Mac), do not require user account and do not need installation of plug-ins.

## Authors' contributions

LR and MP designed the database concept and participated in its design and construction, including the server. MP acted as the final editorial authority. All authors have read and approved the final manuscript.
